# Cadmium-Induced Oxidative Stress: Focus on the Central Nervous System

**DOI:** 10.3390/antiox9060492

**Published:** 2020-06-05

**Authors:** Jacopo J. V. Branca, Claudia Fiorillo, Donatello Carrino, Ferdinando Paternostro, Niccolò Taddei, Massimo Gulisano, Alessandra Pacini, Matteo Becatti

**Affiliations:** 1 Department Experimental and Clinical Medicine, Anatomy and Histology Section, University of Firenze, 50134 Firenze, Italy; jacopojuniovalerio.branca@unifi.it (J.J.V.B.); donatello.carrino@unifi.it (D.C.); ferdinando.paternostro@unifi.it (F.P.); massimo.gulisano@unifi.it (M.G.); alessandra.pacini@unifi.it (A.P.); 2 Department of Experimental and Clinical Biomedical Sciences “Mario Serio”, University of Firenze, 50134 Firenze, Italy; niccolo.taddei@unifi.it (N.T.); matteo.becatti@unifi.it (M.B.)

**Keywords:** cadmium, oxidative stress, mitochondria, ROS, central nervous system, neurovascular unit

## Abstract

Cadmium (Cd), a category I human carcinogen, is a well-known widespread environmental pollutant. Chronic Cd exposure affects different organs and tissues, such as the central nervous system (CNS), and its deleterious effects can be linked to indirect reactive oxygen species (ROS) generation. Since Cd is predominantly present in +2 oxidation state, it can interplay with a plethora of channels and transporters in the cell membrane surface in order to enter the cells. Mitochondrial dysfunction, ROS production, glutathione depletion and lipid peroxidation are reviewed in order to better characterize the Cd-elicited molecular pathways. Furthermore, Cd effects on different CNS cell types have been highlighted to better elucidate its role in neurodegenerative disorders. Indeed, Cd can increase blood–brain barrier (BBB) permeability and promotes Cd entry that, in turn, stimulates pericytes in maintaining the BBB open. Once inside the CNS, Cd acts on glial cells (astrocytes, microglia, oligodendrocytes) triggering a pro-inflammatory cascade that accounts for the Cd deleterious effects and neurons inducing the destruction of synaptic branches.

## 1. Introduction

Cadmium (Cd), a widespread toxic pollutant having a very long biological half-life in humans (20–30 years) and a low rate of excretion from the body, accumulates within human tissues [1]. Cd occupational and environmental exposure comes from metallurgy and the plastic industry, mining, pigments, chemical stabilizers, metal coatings and battery production. Cd-contaminated agricultural land and food are a great source of Cd exposure [2]. Cd can also be found in tobacco smoke, further contributing to human exposure. Prolonged Cd salts accumulation results in harmful effects on several organs, such as the kidney, pancreas, liver, lungs, bones, reproductive organs, and the nervous and cardiovascular systems. Moreover, Cd has been linked to cancer development being classified as category I human carcinogen by the International Agency for Research on Cancer (IARC) [3]. 

Cd exists mainly in the +2 oxidation state and does not directly generate free radicals by oxidation-reduction reactions; however, the indirect formation of reactive oxygen species (ROS) such as superoxide, hydroxyl radical, hydrogen peroxide and nitric oxide has been reported [4,5,6,7].

Basal ROS levels physiologically originate from cell metabolism, including mitochondrial respiratory chain, the reduced form of nicotinamide adenosine dinucleotide phosphate (NADPH)-oxidase and xanthine oxidase [8]. ROS play important roles in the regulation of several physiological processes modulating enzyme activity through redox reaction cycles [9]. When ROS production overwhelms cellular antioxidant defenses, the intracellular redox status can be altered, and oxidative stress, responsible for the harmful effects of Cd, occurs [10]. The mechanisms of Cd-induced oxidative stress are represented by the mitochondrial electron transport chain inhibition, the displacement of redox-active metals, the depletion of antioxidants and the inactivation of antioxidant enzymes [10,11]. 

ROS are reactive molecules/ions originating from oxygen. Some ROS possess unpaired electrons, (free radicals such as superoxide ion (O_2_^•−^) and hydroxyl radical (OH^•−^)), are very unstable and have short half-lives. In contrast, nonradical ROS are stable and with longer half-lives, such as hypochlorous acid (HOCl), hydrogen peroxide (H_2_O_2_), singlet oxygen (^1^O_2_), and peroxynitrite (ONOO^−^). O_2_^•−^ is the precursor of most ROS. It is produced through a one-electron reduction of molecular oxygen and is rapidly transformed into H_2_O_2_ by spontaneous or superoxide dismutase (SOD)-catalyzed dismutation [12]. With regards to other ROS, H_2_O_2_ displays a long half-life, is water-soluble and easily diffuses within and between cells. It accumulates and damages macromolecules through secondarily derived ROS (hypochlorous acid, hydroxyl radical and chloramines). An excessive ROS production induces the oxidation of proteins, lipids, and DNA, causing metabolic pathway alterations and cellular dysfunctions, ultimately leading to necrotic or apoptotic cell death [13]. It is widely accepted that oxidative stress represents the principal molecular mechanism underlying Cd toxicity [4].

## 2. Cd, Mitochondrial Electron Transport Chain and ROS Production

Mitochondria are known as the powerhouses of the cell, converting oxygen and nutrients into chemical energy. An electrochemical proton gradient from the mitochondrial matrix into the intermembrane space is required for the generation of adenosine triphosphate (ATP) from adenosine diphosphate (ADP) and inorganic phosphate. This proton gradient is dependent on the electron transfer down the electron transport chain (ETC) complexes, such as Complex I (NADH–coenzyme Q reductase), Complex II (succinate–coenzyme Q reductase), Complex III (coenzyme Q–cytochrome c reductase) and Complex IV (cytochrome c oxidase) [14]. The metabolic energy from the oxidation of food (sugars, fats, and proteins) is funneled into the formation of reduced coenzymes (NADH) and reduced flavoproteins (FADH_2_). Electrons stored in NADH or FADH_2_ are passed through the ETC complexes that transfer the electrons to molecular O_2_, the terminal acceptor of electrons, reducing it to H_2_O. Each chain component is successively reduced and reoxidized in a redox reaction chain. In this scenario, several steps have the potential to produce potential damaging ROS [15]. The electron transfer from reduced ubiquinone (ubiquinol, QH_2_) to Complex III and the passage of electrons from Complexes I and II to QH_2_ involves the semi-ubiquinone Q^•−^ as an intermediate that can pass an electron to O_2_ generating O_2_^•−^ [16]. It has been shown that about 2% of molecular oxygen is incompletely reduced and converted into O_2_^•−^ in Complex I and Complex III. Mitochondrial ROS formation is favored when mitochondria are not making ATP (for lack of ADP or O_2_) and there is a high NADH/NAD^+^ ratio in the matrix. In these situations, the mitochondrion is under oxidative stress [17]. The mitochondrial ETC is one of the major ROS suppliers in most cells. It has been demonstrated that, in C2C12 myoblasts, 45% of ROS comes from mitochondria [18].

An increasing body of evidence indicates that Cd toxicity may be associated with ROS production at the mitochondrial level [19,20,21,22] but the underlying mechanisms are still unclear. For a long time, it has been known that Cd is responsible for the uncoupling of oxidative phosphorylation [23] and for the inhibition of succinate- and malate/pyruvate-stimulated respiration [24]. Cameron and collaborators suggested that Cd interacts with the Q-site of the Complex I or other NADH-dependent enzymes and not solely exerts an uncoupling action [25]. Physiologically, during the electron transfer produced by NADH-linked substrates, Complex I produces low levels of ROS [26]. However, when the Q-site is inhibited, ROS production by Complex I greatly increases [26]. In addition, when Complex I activity is reduced, the electrons accumulated in the Q-site can be transferred to molecular oxygen resulting in O_2_^•−^ generation [27]. Complex I Fe-S clusters can also be target sites of Cd [28,29]. Furthermore, electrons derived from Complex II can undergo reverse electron transfer to Complex I to generate O_2_^•−^ [30]. Reverse electron transfer occurs when electrons are driven backward through Complex I from reduced ubiquinone to flavin site by high membrane potential maintained by electron flow from succinate at Complex II [31,32,33]. At Complex I flavin site, a direct transfer of electrons to oxygen results in O_2_^•−^ generation [34]. All these data indicate a direct role of Cd in Complex I-derived ROS production.

In 1993, Miccadei and Floridi performed experiments (in isolated rat liver mitochondria) on specific complexes of the respiratory chain showing that Cd inhibited electron flow through Complex III, when two separate ubiquinone reaction centers catalyze the oxidation of ubiquinol to reduce two molecules of cytochrome c, together with the translocation of four protons from the mitochondrial matrix into the intermembrane space. They suggested that Cd is able to block the electron flow rather than uncouple the oxidative phosphorylation [35]. An interesting work in isolated mitochondria from the liver, brain, and heart of guinea pig proposes that Cd induces ROS generation at Complex III [36]. In particular, Cd binding to the Q_o_ site of mitochondrial Complex III prevented electron delivery from semi-ubiquinone to heme b_566_ promoting the accumulation of semi-ubiquinone. The accumulated semi-ubiquinones donate the electron to O_2_, thus forming O_2_^•−^ [36]. The protective effects by stigmatellin, a Complex III inhibitor, against Cd-induced cell damage, confirms the central role of Complex III in Cd-induced ROS formation [37,38].

Recent evidence has also proposed a direct role of Complex II in Cd-induced ROS production [31]. The mitochondrial Complex II, also known as succinate–coenzyme Q reductase, is the smallest mitochondrial ETC complex and, unlike the other respiratory complexes, does not pump protons via the mitochondrial membrane and connects directly the respiratory chain with the tricarboxylic acid (TCA) cycle. When succinate is converted to fumarate in the TCA cycle, FAD is reduced to FADH_2_ and its electrons are transferred to QH_2_ by Fe-S centers. Several mechanisms were proposed to explain the Complex II-mediated ROS generation [39,40,41]. In rat skeletal muscle mitochondria, it was demonstrated that when succinate concentration is low and the oxidation of ubiquinol is inhibited, Complex II can generate high amounts of ROS through the flavin site of the enzyme [40]. Furthermore, in isolated mitochondria, it was showed that high succinate levels associated with a high membrane potential induced reverse electron transfer from Complex II into Complex I with the concurrent enhancement of O_2_^•−^ production [42,43,44]. Hence, it is not unexpected that complex II inhibitors display opposite effects on mitochondrial ROS production, on the basis of energy supply, membrane potential and whole metabolism activity [45,46]. 

The Belyaeva group recently demonstrated the role of mitochondrial Complex II in Cd-induced cytotoxicity. In particular, malonate (Complex II inhibitor) exerted protective effects against Cd-induced necrosis in rat hepatocellular carcinoma AS-30D cells and reduced ROS production induced by Cd in rat pheochromocytoma PC12 cells [31]. 

**Figure 1:**
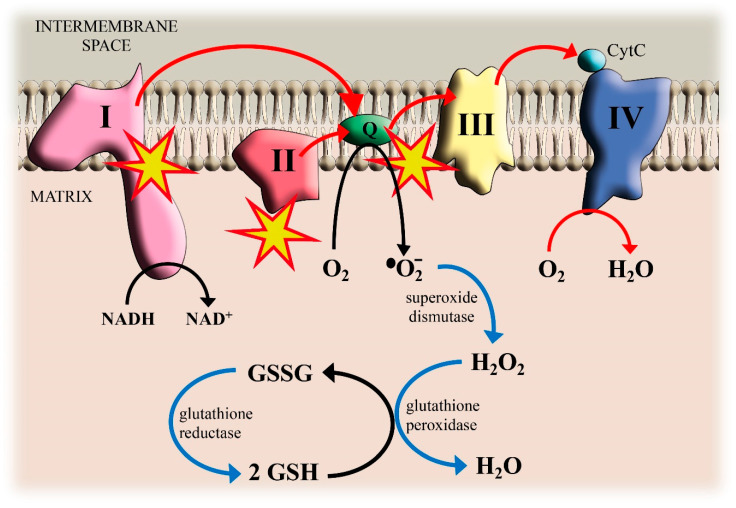
Cadmium (Cd)-induced reactive oxygen species (ROS) formation in mitochondria and antioxidant defenses.

Interestingly, in rat liver isolated mitochondria, different Cd concentrations affected the oxidative phosphorylation in two different ways: low Cd concentration promoted resting state respiration, while high concentrations decreased basal respiration [47]. Moreover, long-term exposure to Cd in breast cancer MDA-MB231 cell line led to an increase in respiration rate without mitochondrial membrane potential variation [48]. On the contrary, short and long-term exposure of immortalized HB2 breast epithelial cells caused an enhancement of respiration rate associated to mitochondrial membrane polarization [49], probably due to a change in energy demand after Cd exposure [50]. Cd-induced cell damage was recently discussed by Al-Ghafari and collaborators on human bone osteoblasts showing electron transport loss and coupling to oxidative phosphorylation, leading to a shift to anaerobic metabolism, with reduced ATP production and oxygen consumption and increased production of lactate [51]. Mitochondrial damage via ROS generation was also demonstrated in human osteoblasts, where a significant enzymes inhibition of mitochondrial respiratory chain and citrate synthase after Cd treatment was observed [20]. It has been accepted that Fe-S clusters of the ETC complexes could be potential Cd targets sites [28,29], resulting in electron transfer inhibition and the dissipation of proton electrochemical gradient necessary for ATP synthesis [29]. These findings were confirmed by Adiele and co-workers that demonstrated the sensibility to Cd of Complexes I, II and III, whereas the nonresponse to Complex IV could be caused by the absence of Fe-S clusters in Complex IV [52]. Furthermore, the excessive ROS production and mitochondrial membrane potential alteration induced by Cd can lead to apoptotic cell death pathways [53]. Cd has also been shown to induce ROS generation by mitochondrial permeability transition pore opening leading to cytochrome c release [54,55]. Since cytochrome c is responsible for the electron transfer from Complex III to Complex IV, the release of cytochrome c disrupts the mitochondrial ETC [56], causing further ROS production [57]. Taken together, these data suggest that mitochondria represent main targets of Cd toxicity but also potential targets for new therapeutic approaches aimed to modulate mitochondrial function (Figure 1).

The electrons transfer (red arrows) from Complexes I and II to QH_2_ and from QH_2_ to Complex III involves the semi-ubiquinone Q^•−^ as an intermediate that can pass an electron to O_2_ generating superoxide O_2_^•−^. The blue arrows show the reactions that detoxify the cell against O_2_^•−^ production. Reduced glutathione (GSH) donates electrons for H_2_O_2_ reduction. GSH is regenerated from its oxidized form (GSSG) by reduction with NADPH. Yellow stars indicate the interaction sites of Cd.

## 3. Cd and Glutathione Depletion

Another important mechanism by which Cd induces oxidative stress is intracellular GSH depletion [58]. The tripeptide GSH is the first line of defense against oxidative stress and is considered to be the major redox buffer in the cell. It is derived from glutamate, cysteine and glycine. GSH biosynthesis is a highly modulated process that occurs in cytosol rather than on ribosomes, in two enzyme-catalyzed, ATP-dependent steps. The major source of GSH synthesis is the liver and, once synthesized, GSH can undergo across the plasma membrane and export from the liver to the bloodstream for supply of other tissues [59]. Some cell types, i.e., energy-ravenous and stress-sensitive brain neurons, are not able to synthesize GSH and import it from adjacent cells of the glia. The oxidized form of glutathione (GSSG), formed during its redox activities, contains two glutathione molecules linked by a disulfide bond. The thiol group in GSH is responsible for its biochemical activity [60]. Reduced GSH is found in millimolar concentrations (1–30 mM) in cellular systems and plays a major role in the detoxification of various electrophilic compounds. It is present in the majority of, if not all, all subcellular compartments [61]. GSH maintains the sulfhydryl groups of proteins in the reduced state and the iron of heme in the ferrous (Fe^2+^) state, and it acts as a reducing agent for glutaredoxin in deoxyribonucleotide synthesis [62]. Moreover GSH has a key role in the synthesis of eicosanoids, steroids and iron-sulfur clusters and modulates oxidative protein folding and redox signaling [63]. GSH is also implicated in transition metals’ chelation, thereby reducing their toxic ability [59], being considered the first line of defense against Cd toxicity [64]. The high affinity of Cd for thiol groups has been demonstrated many years ago in hepatotoxicity induced by Cd [65,66]. In addition, GSH depletion enhances Cd-induced hepatotoxicity and the GSH precursor N-acetylcysteine prevents Cd-induced oxidative stress and toxicity in the liver and brain of Cd pre-exposed rats [67]. These data have been recently confirmed by Zhang and collaborators that demonstrated the protective effects of resveratrol in Cd-induced nephrotoxicity, mitigating GSH depletion and restoring the activity of antioxidant enzymes [68]. Cd toxicity in the brain affects both neurons and glial cells. It has been shown that Cd induces cell death via GSH depletion and ROS production in primary oligodendrocytes [69] and in HT4 mouse neuronal cell line or rat primary mesencephalic cultures [70]. In addition, in primary cortical astroglia cell cultures, Cd treatment depleted GSH levels, leading to intracellular oxidative stress and thiol homeostasis disruption [71]. It has been suggested that Cd-induced GSH depletion may be ascribed to the enhanced activity of gamma-glutamyl transpeptidase enzyme [72] or to the reduced availability of NADPH essential for the activity of glutathione reductase to transform oxidised glutathione (GSSG) to the reduced form (GSH) [73]. Moreover, in rat survival cortical neurons, Cd decreases intracellular GSH levels leading to ROS-mediated cell death [74].

## 4. Cd and Lipid Peroxidation

Oxidative stress can virtually damage all biological macromolecules. Indeed, the oxidized forms of lipids, proteins and DNA are used to assess oxidative damages [75] occurring in most acute and chronic disorders.

Owing to the reactivity of allylic carbons toward ROS, polyunsaturated fatty acids (PUFA) and lipids are highly susceptible to oxidation initiating lipid peroxidation, which, among the mechanisms of damage caused by ROS, is the most extensively studied.

The protonated form of the superoxide anion and the hydroxyl radical are commonly believed to start the autocatalytic lipid peroxidation. Upon hydroperoxyl radical attack at the double bond of (free or phospholipid) PUFA, lipid hydroperoxides are formed through several sequential reactions. Further oxidation of lipid hydroperoxides can cleave the alkyl chain, thus producing a wide variety of lipid-bound aldehydes and ketones and lipid-bound carbonyls, typically termed lipid peroxidation end products [76], responsible for membrane alterations, the efflux of cytosolic solutes and the loss of membrane–protein activities.

Altogether, these reactive electrophiles promptly react with nucleophilic substrates such as amino acid side chains (in particular cysteine, histidine, and lysine), producing stabile adducts. Upon modifications by reactive aldehydes, proteins lose their biological activity or are able to induce inflammatory and immunogenic responses [77,78].

Among the most studied lipoperoxidation products, 4-hydroxy-2-nonenal, malondialdehyde, acrolein and F2-isoprostanes (eicosanoid molecules derived from the peroxidation of arachidonic acid found in biological membranes) represent good biomarkers of oxidative stress [79,80,81] and are measurable in biological fluids. All these molecules have been evoked in the pathogenetic mechanisms of several human disorders, including cancer, cardiovascular and neurological diseases, diabetes and various inflammatory diseases [82,83,84,85]. 

The effect displayed by these biomarkers is evident in biological membranes, which are composed by 70–80% of phospholipids, where fluidity, permeability, and ion transport are significantly altered [86]. Massive lipid peroxidation has been associated with the loss of membrane permeability and cell death. However, whether lipid peroxidation is a cause or effect of cell death is still a matter of debate.

Despite their increasing biological significance, several analytical limitations exist for the quantification of lipid peroxidation products. Currently, High-Performance Liquid Chromatography techniques coupled with mass spectrometry seem to be best suited for their detection and identification [87]. 

Many studies have reported the ability of Cd to alter numerous cellular functions as well as cellular structural components. The effects of Cd have been frequently associated to alteration in redox status usually assessed in terms of cellular GSH levels, activity of antioxidant enzymes, or lipid peroxidation [88,89]. Cd has also been shown to mediate ROS-dependent apoptosis and induce necrotic toxicity, a phenomenon that is strictly linked to the process of lipid peroxidation in multiple organs, including liver, kidney, lungs, and breast [90,91,92]. 

Among the proposed Cd-induced damage mechanisms, lipid peroxidation represents a major consequence of Cd-induced oxidative stress and has been shown to be correlated with the exposure levels to Cd [93]. 

The toxicity of acute exposure to Cd has been evaluated in an animal model assaying plasma redox status in terms of thiobarbituric acid-reactive substance (TBARS) levels. A significant increase in TBARS levels in plasma after one Cd dose (30 mg/kg b.w.) given orally was found [94]. 

In a recent study, evaluating Cd effects on bone, the incubation of human osteoblasts with Cd significantly decreased cell viability in a concentration and exposure-time-dependent manner and induced an enhancement of lipid peroxidation assessed by increased TBARS level [51]. 

In the testis of adult male Wistar rats, the oxidative damaging effect of Cd was revealed by the higher malondialdehyde levels. Interestingly, the results of the study indicate a redox balancing effect, via the reduction of lipid peroxidation, of strawberry methanolic extract on Cd treated testis [95]. 

Upon Cd inhalation, the lungs are the main affected target and develop lung tumors, pulmonary fibrosis and emphysema. Some studies performed in animal models showed that, in response to acute exposition of rats to Cd salts, the evoked inflammatory process may induce a dose-related increase in lung LPO as measured by total lung TBARS levels [96].

In liver injury, cellular and intracellular membranes represent the main targets for Cd, and lipid peroxidation has been considered as an important underlying mechanism. Cellular macromolecule oxidation results in hepatocyte injury, with consequent morphological and functional liver alterations and detrimental effects for the whole organism [97].

In a model of human liver carcinoma (HepG₂ cells), where Cd toxicity was explored in a concentration-dependent manner, an enhancement of lipid peroxidation, witnessed by increased hydroperoxide production, was found specifically at the highest tested Cd concentration. The results of this study clearly prove that Cd treatment induces oxidative stress and apoptosis in human liver carcinoma cells [22]. It has also been shown that the reactive end products of oxidative lipid modifications, such as MDA and 4-Hydroxynonenal (HNE), may enhance the progression of lipid peroxidation and interact with proteins and nucleic acids displaying detrimental effects on these molecules [98].

Cd-induced oxidative stress and cell injury have been recently investigated on human breast cancer cells, which are particularly rich in lipids [99], and, therefore, the incidence of lipid peroxidation has been proposed as a possible mechanism for Cd-induced carcinogenicity [100]. In this study, human breast cancer cells exposed to Cd showed increased levels of lipid hydroperoxides whose cytotoxicity has been attributed to different mechanisms: the modification of the assembly, composition, structure, and dynamics of lipid membranes and the generation of ROS able to crosslink DNA and proteins [101].

Regarding neurological disorders, it was preliminarily reported that occupational exposure to Cd is associated with cognitive impairment in adults [102]. Another study indicated that Cd can be a potent neurotoxic agent for the peripheral nervous system [103]. It was found that, in old men, polyneuropathy was related to the level of the Cd body burden as reflected by urinary Cd, suggesting a key role of Cd in the development of peripheral polyneuropathy at older age [103].

In the experimental animal model, several and heterogeneous neurological pathologic effects have been described in association with lipid peroxidation in some brain regions of developing rats exposed to Cd [104]. Due to its redox-mediated mechanisms, Cd has been indicated as a possible etiological factor for human neurodegenerative diseases, such as Parkinson’s disease, Alzheimer’s disease, and Huntington’s disease [105].

Cd also induces the loss of membrane functions by stimulating the formation of metallothioneins and ROS, thus causing oxidative damage to erythrocytes and various tissues [106]. In rat erythrocytes, the potential protective effects of grape seed proanthocyanidins against Cd-induced oxidative stress has also been evaluated. Lipid peroxidation markers were determined in order to establish erythrocyte membrane oxidative damage. The main results of the study indicate that, upon Cd exposure, the activity of enzymatic and non-enzymatic markers in erythrocytes were reduced and lipid peroxidation markers were increased. Interestingly, grape seed proanthocyanidins displayed protective effects against Cd-induced oxidative stress and lipid peroxidation in Cd-treated rats [107].

Another interesting study revealed that chronic exposure to Cd increased lipid peroxidation and caused the inhibition of antioxidant enzymes inducing oxidative damage in the liver, kidney, and testes [108].

## 5. Cadmium and the Central Nervous System

Cd toxicity can induce cell injury, cell death as well as organ failure by different molecular pathways, mainly including oxidative stress in many body compartments and tissue [109,110,111,112,113,114,115,116,117,118,119], including the central nervous system (CNS) [120]. In this regard, many in vivo and in vitro research studies provided data highlighting the Cd-induced neurotoxicity on the CNS [120] and we revise the Cd role on different compartments and cells among the CNS.

### 5.1. Cadmium and Blood–Brain Barrier

Within the CNS, the blood–brain barrier (BBB) is the specialized system that protects the brain and the spinal cord from harmful and toxic substances and supplies tissues with nutrients. The BBB ability in regulating molecular traffic and keeping out toxins is essential to preserve longevity, health and the integrity of CNS tissues as well as the neural network connectivity [121]. Due to its high importance, BBB dysfunction is implicated in numerous neurodegenerative disorders [122,123].

Such a selective permeable layer is predominantly composed by endothelial cells characterized by tight intercellular junctions that seal the interendothelial space forming a continuous endothelium.

When Cd is present in the blood stream, it can enter the cells by channels, transporters and receptors [124] that are present on the luminal surface of the BBB endothelial cells [125]. Once inside, Cd induces an oxidative stress response that in turn elicits an increase in the antioxidant enzymes’ activity [104]. As a result of the sustained antioxidant defense activity, a widespread depletion of free radical scavenging enzymes (i.e., superoxide dismutase (SOD), glutathione peroxidase (GPx), catalase (CAT), glutathione reductase (GR)) has been observed [126]. Interestingly, Viane and colleagues evidenced that, during Cd acute exposure, most of the CNS is protected from a rapid Cd entry due to the presence of the BBB [127]. On the other hand, chronic and prolonged Cd exposure affects BBB permeability mainly due to the weakening of the cellular antioxidant defenses [126] that, in turn, allow more Cd entering the brain.

In vivo studies clearly showed the ROS involvement in matrix metalloproteinase (MMP) gene activation [128]. These proteolytic enzymes play a key role in the initial opening of the BBB as they disrupt the cerebral vessels tight junctions (TJs) [129].

The importance of Cd-induced oxidative stress in BBB was confirmed by many in vitro analysis. Tobwala and colleagues clearly demonstrated a ROS increase and the consequent GSH activity enhancement after 1 h of Cd treatment in immortalized human brain microvascular endothelial cells (hCMEC/D3) [130]. Upon Cd treatment, the same authors observed neither a significant change in the integrity of the endothelial cell monolayer, as measured by dextran cell-permeability and Trans-Endothelial Electric Resistance (TEER) assays, nor a downregulation of ZO-2 TJ protein evaluated by Western blotting analysis. Accordingly, with Tobwala and colleagues, recently, some authors demonstrated no Cd-dependent ZO-1 TJ protein expression alteration in a rat brain endothelial cell (RBE4) line. Nevertheless, the same authors clearly demonstrated an altered distribution of the ZO-1 TJ as well as the formation of F-actin stress fibers and an abnormal vimentin localization upon Cd administration [131].

### 5.2. Cadmium and Pericytes

Pericytes (PCs), so called due their role of surrounding brain endothelial cells in the perivascular space, are strictly associated to the brain microvessels through elongated processes at the basal lamina level and establish cell-to-cell contacts with endothelial cells by gap junctions. Moreover, they play a pivotal role in maintaining the vascular structure formation and function during cerebrovascular maturation, homeostasis and disease. Indeed, PCs are required for an appropriate brain vascularization at the embryonic stage, predominantly by stabilizing the new vessels’ formation [132] and, during the early postnatal stage, by inducing BBB functional properties [133]. Their functional role is played in cooperation with the nearby cells thus being pivotal in the neurovascular unit (NVU), an assorted structure composed of different cell types, including PCs, extracellular matrix proteins setting up the basal lamina, astrocytes, microglia and neurons [134].

Despite PCs first being characterized at the end of 19th century by Eberth and Rouge, and named at the beginning of 20th century by Zimmermann, very little is known about their functions and their role in brain diseases, also as a potential therapeutic target [135].

PCs play an important role in the maintenance and homeostasis of the BBB [136] and are especially vulnerable to oxidative stress [137]. Indeed, PCs’ oxidative stress has been studied in diabetic mice, highlighting the role of mitochondrial carbonic anhydrases [138]. Similar results were obtained by in vitro experiments with human retinal PCs [139], suggesting, again, the role of mitochondria in oxidative stress generation [140].

Another interesting contribution of PCs in NVU function is linked to the MMP expression particularly induced by inflammatory signaling cascades [141]. In this regard, since activated MMPs have been observed to co-localize with ROS in ischemic brain regions at the level of the capillary walls and astrocytic processes [128], it can be assumed a key role of PCs in regulating BBB permeability during both physiological and deleterious mechanisms.

Furthermore, considering that Cd-induced damage is essentially an oxidative damage, it can be assumed that the Cd-dependent BBB alteration may also involve PCs. This is also confirmed by the presence of the Voltage-Gated Calcium Channels (VGCCs) of l-Type on the PCs’ plasmatic membrane already shown to be involved in the Cd entry into the cells [142,143].

### 5.3. Cadmium and Astrocytes

Carrying on within the NVU scenario, another leading role in this structure was highlighted by astrocytes, serving as the “bridge” between brain microvessels and neuronal synapses [144].

Once Cd induces BBB disruption and permeability increase, it can penetrate and accumulate in the CNS, thus leading to cerebral damage [131].

In vitro studies have demonstrated that Cd toxicity on mouse astrocytes is mediated by GSH depletion as well as oxidative stress [71]. Similar data were obtained on rat astrocytes, where a Cd-dependent rapid increase in ROS production as well as a mitochondrial impairment leading to cell death was evidenced [145]. Interestingly, the widely distributed antioxidant phenolic compounds protect astrocytes against Cd-induced cell death [146].

Recently, in vivo experiments on mice highlighted Cd role in increasing the expression of the glial fibrillary acidic protein (GFAP), an astrocyte marker, both in the cortex and hippocampal regions [147]. Moreover, Cd-induced ROS production was able to modulate the activity of NF-κB, a pleiotropic transcriptional factor involved in cellular responses to many stimuli [148]. Indeed, as demonstrated by Khan and colleagues, the administration of caffeine, a well-known antioxidant molecule and ROS scavenger [149], reduced the phosphorylated-NF-κB levels, suggesting its neuroprotective role in Cd-treated mice via the NF-κB-dependent pathway [147].

This molecular pathway was also supported by Phuagkhaopong and colleagues in in vitro results demonstrating the release of proinflammatory cytokines mediated by the NF-κB pathway from cultured human astrocyte that underwent to Cd accumulation at non-toxic concentration [150]. Moreover, Cd role in oxidative stress induction was confirmed by the effectiveness of curcumin, a new emerging antioxidant compound, in reverting the increase in Cd-dependent ROS production, lipid peroxidation, and NF-κB activation [151]. It is worth noticing that Ndzvetsky and colleagues observed a decrease in glucose-6-phosphate dehydrogenase (G6PD) expression, speculating that Cd can inhibit primary astrocyte viability through glycolysis disturbance, since G6PD plays a crucial role in metabolic energy, mitochondrial activity and is required for antioxidant defense [152].

### 5.4. Cadmium and Microglia

Microglial cells are considered the primum movens within neurological disorders, serving as sentinels capable in orchestrating inflammatory response thanks to their morphological and functional features [153].

Very little is known about Cd effects and oxidative stress induction on microglial cells. One of the first mentions of this derived from the results obtained in primary rat mid-brain neuron-glia cultures showing a Cd-dependent increase in ROS as well as NF-κB overexpression [154]. Furthermore, this study highlighted the increase in metallothioneins, an important piece of antioxidant machinery well known as a heavy-metal-binding protein that participates in protective stress responses [155].

These results are in accordance with Khan and colleagues, who investigated the role of Cd on the BV-2 microglial cell line, evaluating the expression of NF-κB and observing the overexpression of the ionized calcium-binding adapter molecule 1 (Iba1) microglia marker [147].

ROS production was also found in aged rats simultaneously with an intensified staining of the Iba1 marker and an overexpression of the proinflammatory gene CD86 [156]. All these data support the oxidative stress theory of ageing, namely the close interconnection among oxidative stress, ageing and disease [157]. Furthermore, von Leden and colleagues hypothesized that, in aged tissues, microglia is chronically in a pro-inflammatory state [156].

The link between oxidative stress and inflammation is very close; indeed, it has been proven that lipopolysaccharide (LPS) is able to increase cytokines release [158] as well as to induce ROS overproduction [159].

Finally, considering that microglia and astrocytes are in a mutual crosstalk state, [160] a very intriguing scenario is created: oxidative stress induces astrocytes to release pro-inflammatory cytokines that, in turn, activate microglia cells [161,162].

### 5.5. Cadmium and Neurons

One of the first evidence about Cd-induced oxidative stress and its consequences on neurons was found on HT4 cells, a mouse neuroblastoma-derived cell line. Cd treatment on HT4 cells increased protein ubiquitination and the recruitment of the ubiquitin-proteasome machinery for protein degradation [163].

However, it can be assumed that if misfolded proteins are not efficiently degraded, their accumulation contributes to the decrease in cell viability. This suggestion might be in accordance with the results of Wang and colleagues, indicating an endoplasmic reticulum (ER) stress elicited by the increase in the chaperone GRP78 (BiP) that leads to autophagy activation [164]. Indeed, if on the one hand the ER promptly responds to misfolded proteins [165], on the other hand their uncontrolled accumulation in the ER lumen is responsible for the induction of human pathologies, including neurodegenerative disorders [166].

Another important role was assigned to mitochondria whose membrane potential break-down has been hypothesized to be the ROS-induction mediator [74]. These intriguing data were confirmed by in vitro data on cortical neurons from fetal rats and on SH-SY5Y human neuroblastoma cell line showing a Cd-dependent decrease in mitochondrial membrane potential, swelling and cristae loss, as well as cytoplasmic organelles break-down; these in vitro data were corroborated with in vivo data, showing a decreased mitochondrial membrane potential and ROS overproduction [167,168]. Mitochondrial ROS production has also recently been confirmed in PC12 cells [169].

The molecular pathway that triggers ROS production was characterized by Pulido and colleagues who analyzed three different rat hippocampal regions. They concluded that Cd-induced neuronal toxicity was directed by the mitogen-activated protein kinase (MAPK) pathway combined with the mammalian target of rapamycin (mTOR) activity, leading to ROS production and affecting the mitochondrial membrane potential [170,171].

Another aspect to consider that could explain why Cd is counted among the etiopathogenetic factors of degenerative diseases is the Cd-dependent downregulation of growth associated protein-43 (GAP-43) and βIII-tubulin in differentiated SH-SY5Y cell line [172,173]. Since neurodegenerative disorders are associated with synaptic dysfunction [174], the downregulation of these two proteins, highly expressed in the neuronal growth cones throughout development and axonal regeneration, could explain, at least in part, why Cd intoxication could trigger signaling pathways that induce neuronal degeneration.

Finally, to exacerbate the role of Cd-induced oxidative stress, a mention of the antioxidant molecules used to retrieve its detrimental effects is needed. Indeed, many molecules with well-known antioxidant properties were studied to counteract Cd-induced neural oxidative stress, such as royal jelly [175], flavonoids and polyphenols content in *Fragaria ananassa* methanolic extract [176], selenium [172,177], zinc [172] and CBD [173] as new emerging antioxidant tools in neurodegenerative disorders [178]. Furthermore, in order to counteract the Cd deleterious effects, some studies highlighted the role of divalent metal ions such as magnesium, calcium, zinc in competing with Cd for the same binding sites [179,180,181].

### 5.6. Cadmium and Oligodendrocytes

Oligodendrocytes are myelin-enriched cells essential to insulate neurons, thus maintaining proper brain functions. However, oligodendrocytes are very susceptible to oxidative stress and demyelination may occur from brain disorders [69].

Very little is known about Cd-induced oxidative stress on oligodendrocytes. The main research studies linked the Cd-induced toxic effects on ROS production and its modulation by GSH levels [69]. A deeper study has been conducted by Hossain and colleagues that defined an intricate molecular pathway of Cd-induced apoptotic cell death entailing ROS generation, Bax translocation and the consequent cytochrome C spillage from mitochondria, thus leading to cell death [182].

Concerning the high lipid content within the myelin sheets, lipid peroxidation may have a pivotal role in demyelination allowing oligodendrocytes to be vulnerable. In addition, oligodendrocytes express low levels of antioxidant molecules, thus contributing again to susceptibility to oxidative stress [183].

Another interesting scenario arose from the cross-talk between microglia and oligodendrocytes as previously extensively reviewed [184], as well as by astrocyte-derived cytokines [185]. Cytokines are important in immune response coordination and their dysregulation plays a key role in neuroinflammation, neurodegeneration, and demyelination as well [186].

In this regard, it has been demonstrated that in LPS-activated microglia a cytokine release, an overexpression of inducible nitric oxide synthase, as well as a ROS production increase occur together with demyelination and axonal damage in cerebellar cultures [158].

Nevertheless, both microglia and astrocytes can direct oligodendrocyte remyelination [187], especially following the application of M2-activated microglia (the anti-inflammatory/immunoregulatory functional phenotype) conditioned medium [188], and through the secretion of factors that stimulate the recruitment of oligodendrocyte progenitor cells.

## 6. Conclusions

**Figure 2:**
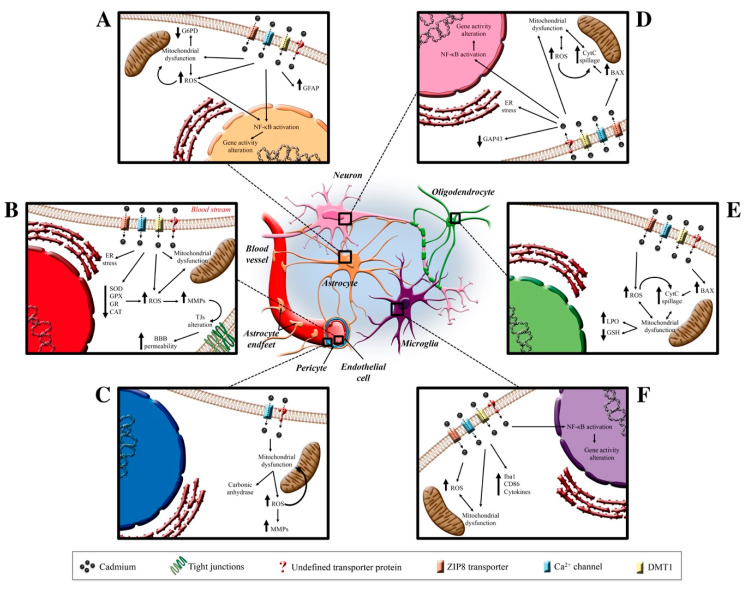
Cd effects on neurovascular unit. In the center of the figure, the CNS protagonists are illustrated who are the target of Cd-induced neurotoxicity. In Insert (**A**) (top left), the putative channels and transporters through which Cd enters the astrocytic cells are reported (ZIP8 transporters, Ca^2+^ channels, DMT1, and undefined transporter). Once inside, Cd elicits ROS production that causes a mitochondrial disfunction and, in turn, induces the NF-κB activation and a consequent gene transcription alteration. Insert (**B**) (middle left) shows the known Cd-dependent molecular pathway involving a decrease in antioxidant systems and ER stress that induces an MMP alteration and a TJs disassembly-dependent BBB alteration. Insert (**C**) (lower left) shows the Cd-dependent alterations in pericytes. Insert (**D**–**F**) (on the right) summarizes the Cd-dependent alterations that involve neuron, oligodendrocyte and microglia, respectively. Abbreviations: BAX (Bcl-2 associated protein); BBB (blood-brain barrier); CAT (catalase); CD86 (cluster of differentiation 86); CytC (cytochrome C); ER (endoplasmic reticulum); G6PD (glucose-6-phosphate dehydrogenase); GAP43 (growth associated protein 43); GFAP (glial fibrillary associated protein); GPX (glutathione peroxidase); GR (glutathione reductase); GSH (glutathione); Iba1 (ionized calcium-binding adapter molecule 1); LPO (lipid peroxidation); MMPs (matrix metalloprotease); NF-κB (nuclear factor kappa-light-chain-enhancer of activated B cells); ROS (reactive oxygen species); SOD (superoxide dismutase); TJs (tight junctions).

Cd is widespread in our environment and in our lives. Among its deleterious effects in many organs and tissues, it plays a crucial role in CNS, reaching the BBB and increasing its permeability, allowing for, in turn, a growing entrance in the brain. Once inside, Cd triggers a plethora of molecular pathways (Figure 2) and, in this regard, oxidative stress has been observed to play multiple roles in Cd-mediated toxicity, including ROS production, mitochondrial ETC inhibition and GSH depletion. While the initial adaptive antioxidant response to chronic, low-dose Cd exposure can reduce ROS production, oxidative damage may accumulate over time, leading to cell damage.
